# The exploration of B cell maturation antigen expression in plasma cell dyscrasias beyond multiple myeloma

**DOI:** 10.1186/s12885-023-10591-1

**Published:** 2023-02-07

**Authors:** Yanjie Xu, Xia Mao, Yimei Que, Menglei Xu, Chunhui Li, Varlene Daniela Fernandes Almeida, Di Wang, Chunrui Li

**Affiliations:** 1 Department of Hematology, Tongji Hospital of Tongji Medical College, Huazhong University of Science and Technology, 1095 Jie-Fang Avenue, Wuhan, Hubei 430030 P. R. China; 2 Immunotherapy Research Center for Hematologic Diseases of Hubei Province, Wuhan, 430030 Hubei China; 3 Tongji Medical College, Huazhong University of Science and Technology, Wuhan, 430030 Hubei China

**Keywords:** BCMA, Immunotherapy, Plasma cell dyscrasia, Multiple myeloma, Amyloidosis

## Abstract

**Background:**

B cell maturation antigen (BCMA) targeted immunotherapies have demonstrated remarkable clinical efficacy in multiple myeloma (MM). Here, we evaluated the BCMA expression in MM and other plasma cell dyscrasias (PCDs), hoping to provide a potential treatment strategy for the relapsed/refractory PCDs besides MM.

**Methods:**

From January 2018 to August 2021, 377 patients with PCDs were enrolled in this study, including 334 MM, 21 systemic light chain amyloidosis (AL), 5 POEMS syndrome, 14 monoclonal gammopathy of undetermined significance (MGUS), and three monoclonal gammopathy of renal significance (MGRS). The membrane-bound BCMA expression measured by multiparameter flow cytometry was defined by BCMA positivity rate and the mean fluorescence intensity (MFI).

**Results:**

The patients with MM had a median BCMA positive rate of 88.55% (range, 0.2% - 99.9%) and median BCMA MFI of 1281 (range, 109 - 48586). While the median BCMA positive rate in other PCDs was 55.8% (6.2% -98.9%), and the median BCMA MFI was 553 (182- 5930). BCMA expression level was negatively associated with hemoglobin concentration in multivariate analysis in terms of BCMA positive rate and MFI.

**Conclusions:**

In conclusion, BCMA has the potential to be a therapeutic target for other PCDs besides MM.

**Supplementary Information:**

The online version contains supplementary material available at 10.1186/s12885-023-10591-1.

## Introduction

B cell maturation antigen (BCMA), also known as CD269, is a cell-surface receptor that belongs to the tumor necrosis factor receptor superfamily. It has been reported to be exclusively expressed by plasma cells and mature B lymphocytes [1–4]. There is an increased expression of BCMA in the malignant plasma cells compared with normal plasma cells [5]. A recent study reported that BCMA contributes to myeloma cell survival and growth in vivo and in vitro [6]. These characteristics suggest that BCMA is an ideal therapeutic target for multiple myeloma (MM) and make it the first antigen studied in clinical trials as a chimeric antigen receptor (CAR) target. The BCMA-targeted clinical trials demonstrated dramatic efficacy and safety profiles in refractory/relapsed MM [7, 8].

Plasma cell dyscrasias (PCDs) are a group of heterogeneous disorders characterized by the proliferation of monoclonal plasma cells. The clonal plasma cell burden is relatively low in some PCDs, including systemic light chain amyloidosis (AL), POEMS syndrome, monoclonal gammopathy of undetermined significance (MGUS), and monoclonal gammopathy of renal significance (MGRS) [9–12]. The treatment algorithm for PCDs that need intervention is empirical and often derived from MM. For example, the mainstay of therapy remains to target the clonal plasma cells to reduce the synthesis of the amyloid light chain in patients with AL [13]. The same strategies were used for POEMS syndrome [11]. Therefore, we wonder if the success of BCMA-targeted therapy in MM could be translated to other PCDs to improve the clinical outcome. There has been an instance of applying BCMA-CAR T cell therapy in our center on a patient with POEMS syndrome, who achieved a stringent complete remission with grade 1 cytokine release syndrome [14]. To further explore such a possibility, we evaluated the expression of BCMA in patients with PCDs in this study.

## Methods

### Patients

With the approval of the medical ethics committee of Tongji Medical College, Huazhong University of Science and Technology, Wuhan, China, we conducted this retrospective study. From January 2018 to August 2021, 377 patients were enrolled in this study with available flow cytometry examination of BCMA expression in our hospital. The patient population consists of 334 MM, 21 AL, 14 MGUS, 5 POEMS, and 3 MGRS.

The diagnosis of MM, MGUS, AL, and POEMS syndrome was confirmed according to the International Myeloma Working Group (IMWG) updated criteria [15]. The diagnosis of MGRS was confirmed according to the International Kidney and Monoclonal Gammopathy Research Group (IKMG) diagnostic criteria [12].

### Flow cytometric analysis of BCMA expression

The level of membrane-bound BCMA expression was defined by BCMA positivity rate, the percentage of BCMA positive clonal plasma cells within the bone marrow plasma cells compartment, and the BCMA expression intensity, which was determined by quantitative flow cytometric measurements based on the mean fluorescence intensity (MFI) of the whole monoclonal plasma cell population.

Surface antigen BCMA expression was detected by multiparameter flow cytometry (FACScanto, Becton Dickinson). BD FACSDiva 8.0.2 software was used for the data analysis. And the fluorochrome-conjugated anti-human antibodies were purchased from BD Diagnostics.

A panel of flow cytometry antibodies was used to detect clonal plasma cells: a cluster of differentiation CD38, CD138, CD45, CD19, CD56, CD20, CD22, CD117, intracytoplasmic immunoglobulin light chain (clambda, ckappa). The initial analysis gate was devised using a side scatter histogram and CD38 expression. The plasma cells compartment was identified by CD38 positive and SSC medium expression, usually with the concurrent CD138 positive expression. The clonal plasma cells were distinguished from normal plasma cells by aberrant phenotypic profiles, such as CD56 positive and CD19 negative. When the plasma cells showed a phenotype of CD56 negative, additional markers like clambda/ckappa, CD45, CD20, and CD117 would be required. The detailed gated method can be seen in Figure S1.

### Statistical analysis

Analysis was performed using GraphPad Prism version 8. Categorical variables were described as frequencies (percentages), parametric data were presented as means (± standard deviation, SD), and nonparametric data were described as medians (ranges). The Mann-Whitney U test was used to compare two groups for quantitative variables, and the Kruskal-Wallis test with Dunn’s multiple comparison test was used for three or more groups. The D'Agostino-Pearson normality test was performed to verify the distribution of the data. The correlation analysis was performed according to Spearman’s correlation test. Multivariate linear regression analysis was applied to analyze the association of clinical parameters with BCMA expression. All the tests were two-tailed, and the *P*-value of less than 0.05 was taken as statistically significant. Statistical significance was regarded as *p<* 0.05 (*), *p<* 0.01 (**), *p<* 0.001 (***), and *p<* 0.0001 (****).

## Results

### BCMA expression in plasma cell dyscrasias

**Fig. 1:**
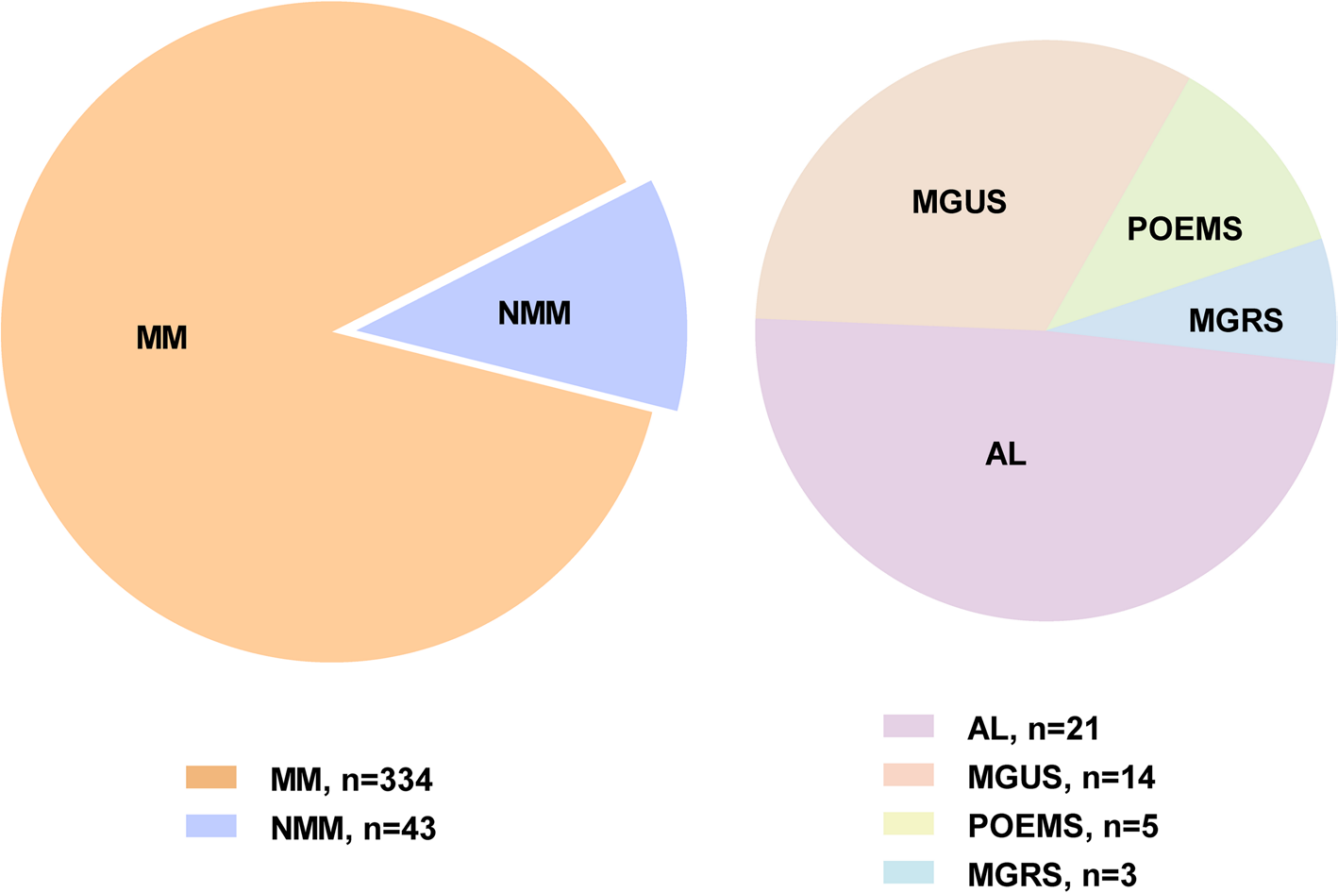
The proportion of patients in each diagnostic category. A total of 377 inpatients were included in this study, in which there were 334 patients with MM, 21 patients with AL, 14 patients with MGUS, five patients with POEMS syndrome, three patients with MGRS. Abbreviations: MM, multiple myeloma; NMM: non-MM, including AL, MGUS, POEMS syndromes, and MGRS. AL, systemic light chain amyloidosis; MGUS, monoclonal gammopathy of undetermined significance; MGRS, monoclonal gammopathy of renal significance

A total of 377 inpatients were enrolled in this study. The MGUS, MGRS, AL, and POEMS syndrome were grouped as NMM (non-MM). And the pie chart shows the distribution of patients in each diagnostic category in Fig. 1. The clinical characteristics of patients with MM and NMM are presented in Table 1. Histopathological findings on the MGRS patients are shown in Figure S2. Two of these three MGRS patients had renal biopsy before they came to our hospital, whose original pathology data were unavailable.

**Table 1:** Clinical characteristics of patient population

characteristic	NMM (*n=*43)	MM (*n=*334)	*P* value
Median age (range), years	56 (34 -78)	60.5 (26 - 91)	0.01*
Sex (male), (n, %)	32 (74.42%)	199 (59.58%)	0.06
Median hemoglobin (range) (g/L)	127 (81 - 162)	89 (36 - 166)	< 0.0001*
Median serum albumin (range) (g/L)	37.60 (14.90 - 49.50)	35.3 (14.10 - 51.60)	0.99
Median serum LDH (range) (U/L)	201 (86 - 426)	190.5 (57 - 1867)	0.20
Median serum calcium (range) (g/L)	2.37 (2.13 - 2.78)	2.47 (1.91-4.40)	0.0003*
Median serum creatinine (range) (g/L)	84 (43 - 583)	89 (36 - 1210)	0.33
Median serum β2-M (range) (g/L)	2.9 (1.44 - 14.53)	5.37 (1.49 - 80)	< 0.0001*
M protein isotype			0.16
IgG	18 (41.86%)	142 (42.51%)	
IgA	14 (32.56%)	89 (26.65%)	
Light chain	7 (16.28%)	70 (20.96%)	
IgD&IgE	0	21 (6.29%)	
others	4 (9.30%)	12 (3.59%)	
Prior therapy			
Auto-HSCT	0	16 (4.79%)	0.287
BCMA CART trial	0	13 (3.89%)	0.383

*MM* Multiple myeloma, *NMM* Non-MM, the group including systemic light chain amyloidosis, monoclonal gammopathy of undetermined significance, monoclonal gammopathy of renal significance, and *POEMS* Syndromes, *M* Protein, monoclonal protein, *LDH* Lactic dehydrogenase, *β2-M* β2-microglobulin, *Auto-HSCT* Autologous hematopoietic stem cell transplantation

^*^: *p* values less than 0.05 (two-tailed) were considered statistically significant

**Fig. 2:**
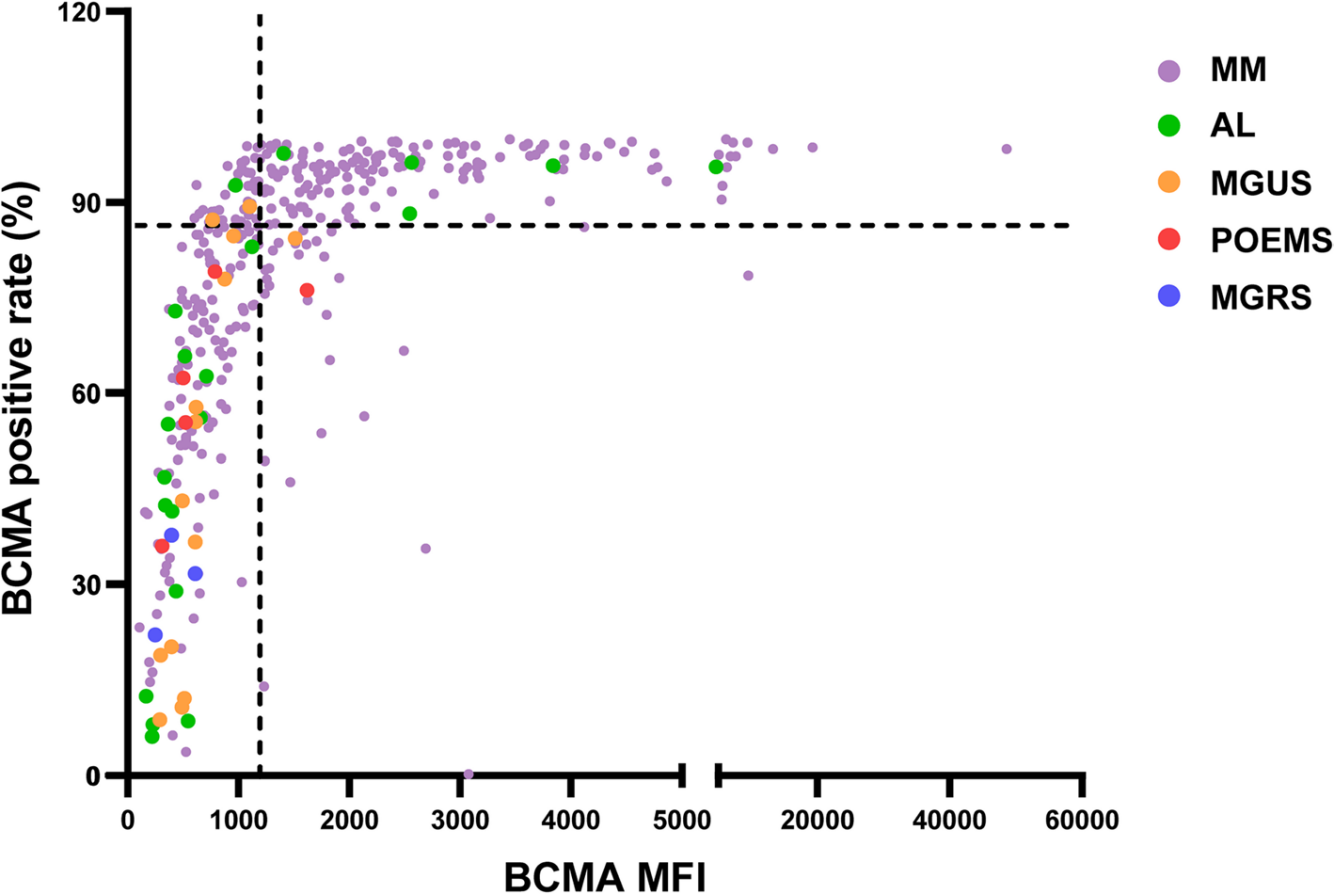
Overview of the expression of BCMA in plasma cell dyscrasias. Scatterplot depicted the BCMA expression in all the plasma cell dyscrasias according to BCMA positive rate and MFI. It was divided into four quadrants by median lines. Different colors of the dots indicated different populations: MM (purple), AL (green), MGUS (yellow), POEMS syndrome (red), MGRS (blue). The BCMA positive rate ranged from 0.2% to 99.9%, with a median of 87.1%, and BCMA MFI ranged from 109 to 48586 with a median of 1181. Abbreviations: BCMA, B cell maturation antigen; MFI, mean fluorescence intensity; MM, multiple myeloma; AL, systemic light chain amyloidosis; MGUS, monoclonal gammopathy of undetermined significance; MGRS, monoclonal gammopathy of renal significance.

The expression of membrane BCMA varied in all of these subjects. BCMA positive rate ranged from 0.2% to 99.9%, with a median of 87.1%, and BCMA MFI ranged from 109 to 48586 with a median of 1181, as shown in Fig. 2. Most patients in the NMM group were located in the left lower quadrant, which represented a relatively lower BCMA positive rate and MFI.

**Fig. 3:**
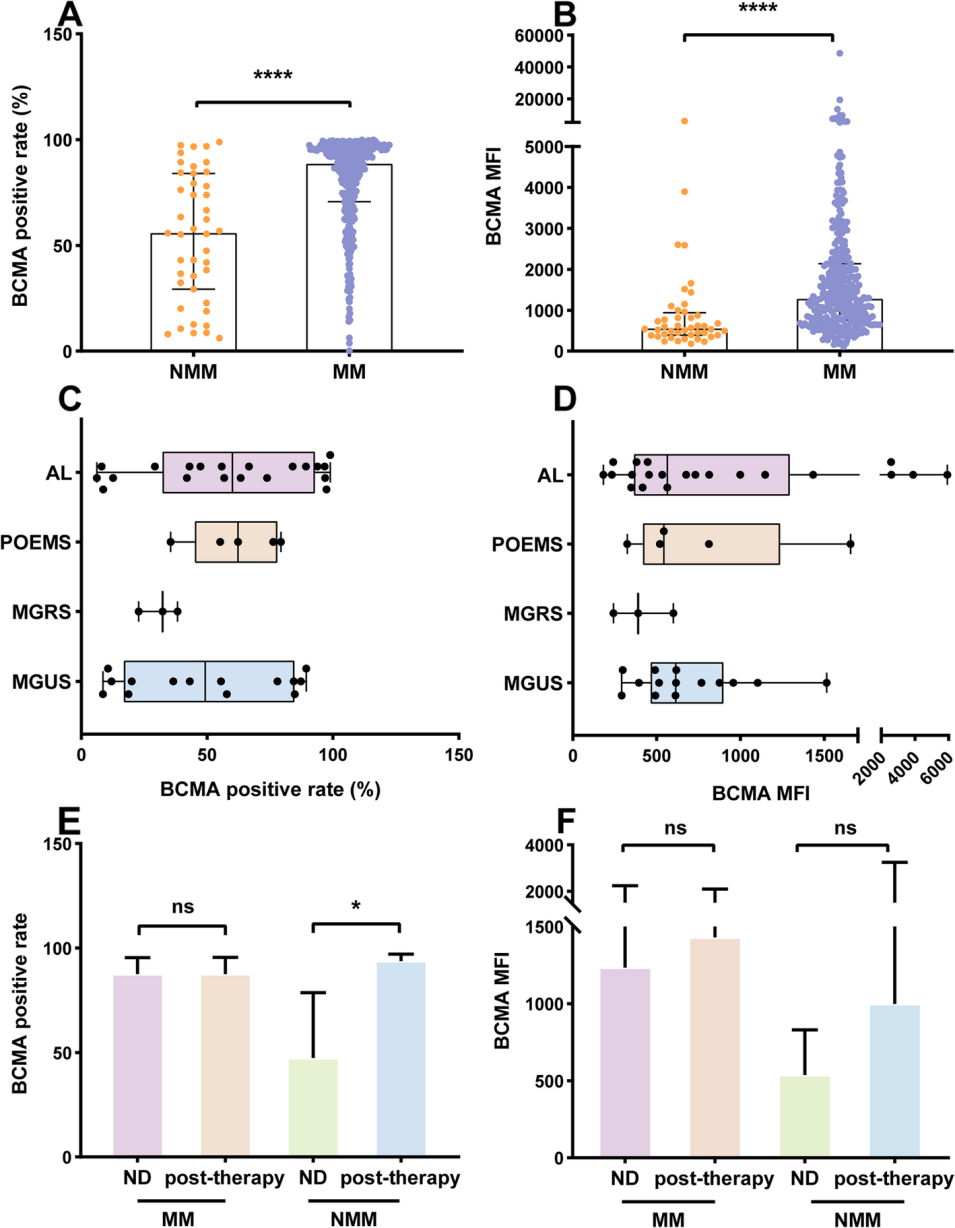
The expression of BCMA in plasma cell dyscrasias. **A**, **B** The bar diagrams showed the BCMA expression rate and BCMA MFI in patients with MM and NMM, respectively. The patients with MM had a significantly higher BCMA expression in both BCMA positive rate and BCMA MFI. The patients with MM had a median BCMA positive rate of 88.55% (range, 0.2% - 99.9%) and median BCMA MFI of 1281 (109 - 48586). While the median BCMA positive rate in NMM patients was 55.8% (6.2% -98.9%), and the median BCMA MFI was 553 (182- 5930). **C**, **D** There were no statistically significant differences between BCMA positive rate and BCMA MFI among patients with AL, POEMS syndrome, MGRS, and MGUS. The median BCMA expression rate in patients with AL, POEMS syndrome, MGRS, MGUS was 60.1% (6.2- 98.9%), 62.3% (35.5- 79.3%), 32.3% (22.8- 38.3%), 49.3% (8.6- 89.4%), respectively. The median BCMA MFI was 563 (182- 5930), 543 (325- 1656), 390 (243- 600), 614 (291- 1514), respectively. **E**, **F** There was no significant difference between the newly diagnosed and previously treated (post-therapy) patients with MM for both BCMA positive rate and MFI. The newly-diagnosed patients (*n=*244) with MM had a median BCMA positive rate of 86.55% (3.7- 99.9%), which was 87.9% (0.2- 99.9%) for the pre-treated (*n=*90) patients. The median BCMA MFI for the newly-diagnosed patients was 1235 (109- 19261) and 1431 (382- 48586) for the pre-treated patients. In the NMM group the median BCMA positive rate for the newly-diagnosed patients (*n=*38) was 47.4% (6.2 – 98.9%), and 93.8% (55.5 - 97.4%) for the pre-treated patients (*n=*5). The pre-treated patients had a higher BCMA expression rate than the newly-diagnosed patients, which may be due to the limited sample size of pre-treated patients. The median BCMA MFI for the newly-diagnosed patients were 538 (182 - 5930) and 998 (446 - 3897) for the pre-treated patients. There was no significant difference in BCMA MFI between newly diagnosed and pre-treated patients

We found that the patients with MM had a significantly higher level of BCMA expression than those with NMM, as detailed in Fig. 3 (A, B). The expression of BCMA in AL, MGUS, MGRS, and POEMS syndrome was exhibited in Fig. 3 (C, D), among which there were no significant differences.

It is worth noting that BCMA expression can still be detected in patients’ post-therapy. 95 out of the 377 patients had received at least one cycle of chemotherapy when tested by flow cytometry. As shown in Fig. 3 (E, F), there was no significant difference between the newly diagnosed and pre-treated MM patients in BCMA positive rate and MFI. As for the NMM group, the pre-treated patients had a higher BCMA expression rate than the newly diagnosed patients, which may be due to the limited sample size of the pre-treated group. At the same time, there was no significant difference in the median BCMA MFI.

### The correlation of BCMA expression level with clinical parameters

**Fig. 4:**
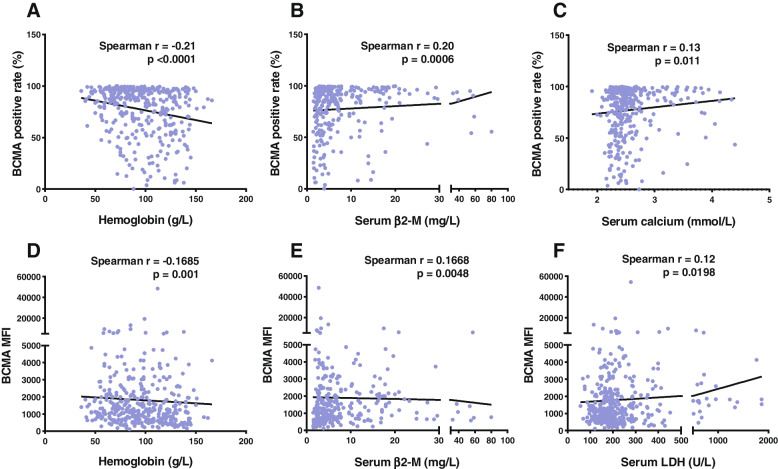
Spearman correlation analysis of BCMA expression and clinical parameters. The correlations with statistically significant *p* values (<0.05) were exhibited. Figures **A**, **B**, **C** showed Spearman correlations between BCMA positive rate and hemoglobin, serum β2-M, and serum calcium, respectively. Figures **C**, **D**, **E** showed the correlations between BCMA MFI and hemoglobin, serum β2-M, and LDH, respectively. Abbreviations: BCMA, B cell maturation antigen; MFI, mean fluorescence intensity; β2-M, β2-microglobulin; LDH, lactic dehydrogenase

We analyzed the correlations between the BCMA expression and clinical parameters in these PCD patients based on the Spearman correlation analysis, which included the history of therapy, gender, age, hemoglobin, albumin, serum calcium, serum creatinine, serum β2-microglobulin (β2-M), lactic dehydrogenase (LDH) auto-HSCT (autologous hematopoietic stem cell transplantation) and BCMA CAR T. There was a significant inverse correlation between BCMA positive rate and hemoglobin. BCMA's positive rate positively correlated with β2-M and serum calcium. As for BCMA, MFI was inversely correlated with hemoglobin either and positively correlated with β2-M and LDH, as detailed in Fig. 4. Though the association between BCMA expression and clinical parameters in these PCD patients was significant, it was hard to form a statistically relevant conclusion since Spearman’s coefficients for all these parameters were less than |0.21|. In newly diagnosed patients, BCMA positive rate was negatively correlated with hemoglobin and positively correlated with β2-M (Table S1).

Multivariate linear regression analyses were performed to evaluate the independent associations between BCMA expression and the clinical factors aforementioned, in which the BCMA MFI value was log10-transformed. In multiple regression analysis, the factors independently associated with BCMA positive rate were hemoglobin and albumin. And only the hemoglobin remained negatively associated with log BCMA MFI (Table 2). Similar results could be seen in newly diagnosed patients (Table S2). BCMA positive rate was associated with hemoglobin and albumin. Age, hemoglobin and LDH were associated to log BCMA MFI though Spearman’s coefficients for all these parameters were less than |0.006|.

**Table 2:** Multivariate linear regression analysis of clinical parameters with BCMA expression

	**BCMA positive rate**			**Log BCMA MFI**		
	**Coefficient**	**95% CI**	***P***	**Coefficient**	**95% CI**	***P***
Gender	-1.209	-7.016 - 4.598	0.682	0.054	-0.034 - 0.142	0.225
Age	-0.071	-0.395 - 0.253	0.667	-0.004	-0.009 - 0.001	0.135
History of therapy	4.435	-3.046 - 11.916	0.244	0.080	-0.032 - 0.191	0.162
Hemoglobin	-0.277	-0.398 - -0.156	< 0.001*	-0.003	-0.004 - -0.001	0.005*
Serum albumin	0.481	0.056 - 0.905	0.027*	0.003	-0.004 - 0.009	0.385
Serum calcium	7.069	-0.223 - 14.362	0.057	0.053	-0.057 - 0.163	0.344
Serum creatinine	0.008	-0.023 - 0.039	0.606	< 0.001	0 - 0.001	0.183
Serum β2-M	-0.144	-0.592 - 0.304	0.528	-0.005	-0.012 - 0.002	0.150
Serum LDH	0.008	-0.005 - 0.02	0.242	< 0.001	-0.0004 - 0.0003	0.113
Auto-HSCT	-1.405	-15.763 - 12.954	0.847	-0.085	-0.295 - 0.126	0.429
BCMA CART trial	-9.582	-26.432 - 7.267	0.264	0.098	-0.145 - 0.34	0.427

*β2-M*, β2-microglobulin, *LDH* Lactic dehydrogenase, *BCMA* B cell maturation antigen, *MFI* mean fluorescence intensity, *Auto-HSCT*, Autologous hematopoietic stem cell transplantation

^*^: *p* values less than 0.05 (two-tailed) were considered statistically significant

## Discussion

Despite the small and relatively “benign” clone, some PCDs, such as AL and POEMS, require therapeutic intervention. Unfortunately, relapsed or refractory diseases are inevitable for these patients, followed by the progression of organ involvement. The severity of organ damage greatly affects patients' quality of life and even survival [13, 16]. To date, there is no consensus on the therapy for these relapsed/refractory diseases [17]. New agents have been introduced and evaluated, one of which is daratumumab, a monoclonal anti-CD38 antibody with an established role in the MM immunotherapy landscape [18–20]. With the remarkable efficacy and manageable safety, daratumumab has been approved by the FDA of US in newly diagnosed AL patients [21, 22]. BCMA has a more favorable expression pattern than that of CD38, which has an extensive tissue distribution, including lymphoid and myeloid cells, as well as the normal tissues of nonhematopoietic origin [23]. Thus, BCMA may be superior to CD38 in terms of on-target off-tumor effect theoretically. The BCMA-targeted therapies have been undergoing extensive development in MM, including antibody drug conjugates, bispecific antibodies, and CAR T cell therapies, all achieved remarkable clinical outcomes [8, 24, 25]. Belantamab mafodotin, an antibody drug conjugate, and Idecabtagene vicleucel, a BCMA-CAR T cell product, have already secured FDA approval for the treatment of relapsed MM [26–28]. These BCMA-targeted therapies seem to be an attractive alternative treatment option for the PCDs with preferable safety. In our study, membrane BCMA was detected in the monoclonal plasma cells in all the subjects at varying levels. Of note, it persisted on the plasma cells during chemotherapies or after relapse. The results demonstrated the possibility for BCMA to be a potential target in the PCDs besides MM. Except for the POEMS syndromes, there also had been a case reporting the first use of BCMA-CAR T therapy in concomitant AL in the setting of MM. The patient achieved hematological stringent complete remission and organ renal response with a decrease of 70% of proteinuria [29]. Therefore, BCMA-targeted therapies have the rationale to be extensively evaluated in the clinical trials for PCDs. It will be more convincing if a comparative assessment on BCMA expression of clonal and non-clonal plasma cells in each disease entity is made. To confirm this, further prospective studies are needed.

In this study, we also found that the level of BCMA expression in the PCDs with low clonal plasma cell burden was relatively lower than that in MM. The PCDs present distinct biological characteristics and different burdens of plasma-cell-derived clone, which suggests that the nature of monoclonal plasma cells may be distinctive [30, 31]. At the level of gene expression, AL is a molecularly distinct entity from MM, and the gene expression profile resembles remarkable similarity of normal plasma cells, rather than myeloma cells [30]. Another study showed that single-cell RNAseq could identify MGUS plasma cells from normal and myeloma plasma cells [30]. Based on this, we speculate that BCMA may present different expression patterns in these PCDs. But researches with a larger study cohort are needed to validate the hypothesis in the future. The relatively low expression of BCMA in the PCDs may raise concerns about antigen-negative relapse or reduced efficacy, though the impact of surface antigen expression level remains uncertain in the BCMA-targeted therapies [32]. In fact, the aforementioned AL case treated with BCMA-CAR T therapy achieved durable response with stringent complete remission, though the BCMA expression rate was 22% [29].

We observed a significant negative correlation between BCMA expression level and hemoglobin concentration in univariate and multivariate analyses. Anemia is a prevalent complication of MM; however, the underlying mechanism has not been completely elucidated. The cause of myeloma-related anemia is considered to be multifactorial in previous studies. The probable pathophysiological mechanisms included impaired iron utilization [33, 34], physical infiltration replacement [35], and insufficient erythropoietin production resulting from renal dysfunction [36]. The upregulation of the apoptogenic receptors on malignant plasma cells' surfaces may also exert direct cytotoxicity on erythropoietic precursors [37]. In addition, the cytokines aberrantly secreted by malignant plasma cells, such as transforming growth factor-β and chemokine CCL3, further impaired the erythroid precursors [38–40]. We speculated that BCMA might be potentially involved with the defective erythropoiesis dependent on or independent of the mechanism above. Few studies focused on the anemia of PCDs with a low plasma cell burden. This finding of the study needs to be verified in future studies

## Conclusions

In conclusion, our study demonstrated that membrane BCMA was generally expressed by plasma cell dyscrasias. Therefore, there is a rationale to translate the BCMA-targeted therapy to other PCDs besides MM. However, the sample size of the NMM group was small, and further studies with a larger study population are required.

## Supplementary Information

**Additional file 1:** **Figure S1**. The detailed gated method of flow cytometry. **Figure S2.** The pathology of MGRS patient. **Table S1.** Spearman correlation analysis of clinical parameters with BCMA expression in newly diagnosed patients. **Table S2.** Multivariate linear regression analysis of clinical parameters with BCMA expression in newly diagnosed patients.

## Data Availability

The original contributions presented in the study are included in the article. Further inquiries can be directed to the corresponding author.

## Abbreviations

BCMA: B cell maturation antigen
MM: Multiple myeloma
PCD: Plasma cell dyscrasia
AL: Systemic light chain amyloidosis
MGUS: Monoclonal gammopathy of undetermined significance
MGRS: Monoclonal gammopathy of renal significance
CAR: Chimeric antigen receptor
MFI: Mean fluorescence intensity
β2-M: β2-microglobulin
LDH: Lactic dehydrogenase
Auto-HSCT: Autologous hematopoietic stem cell transplantation
