# Supplementary material for: The exploration of B cell maturation antigen expression in plasma cell dyscrasias beyond multiple myeloma
Source: BMC Cancer. 2023 Feb 7;23:123. doi: 10.1186/s12885-023-10591-1 (PMC9903528; doi:10.1186/s12885-023-10591-1)
Supplement: Supplementary file 1 — Additional file 1: Figure S1. The detailed gated method of flow cytometry. Figure S2. The pathology of MGRS patient. Table S1. Spearman correlation analysis of clinical parameters with BCMA expression in newly diagnosed patients. Table S2. Multivariate linear regression analysis of clinical parameters with BCMA expression in newly diagnosed patients. [file 12885_2023_10591_MOESM1_ESM.docx]

**CONTENTS:**

**Supplemental Figures**

Figure S1. The detailed gated method of flow cytometry.

Figure S2. The pathology of MGRS patient.

**Supplemental Tables**

Table S1. Spearman correlation analysis of clinical parameters with BCMA expression in newly diagnosed patients.

Table S2. Multivariate linear regression analysis of clinical parameters with BCMA expression in newly diagnosed patients.

**Figure S1.**


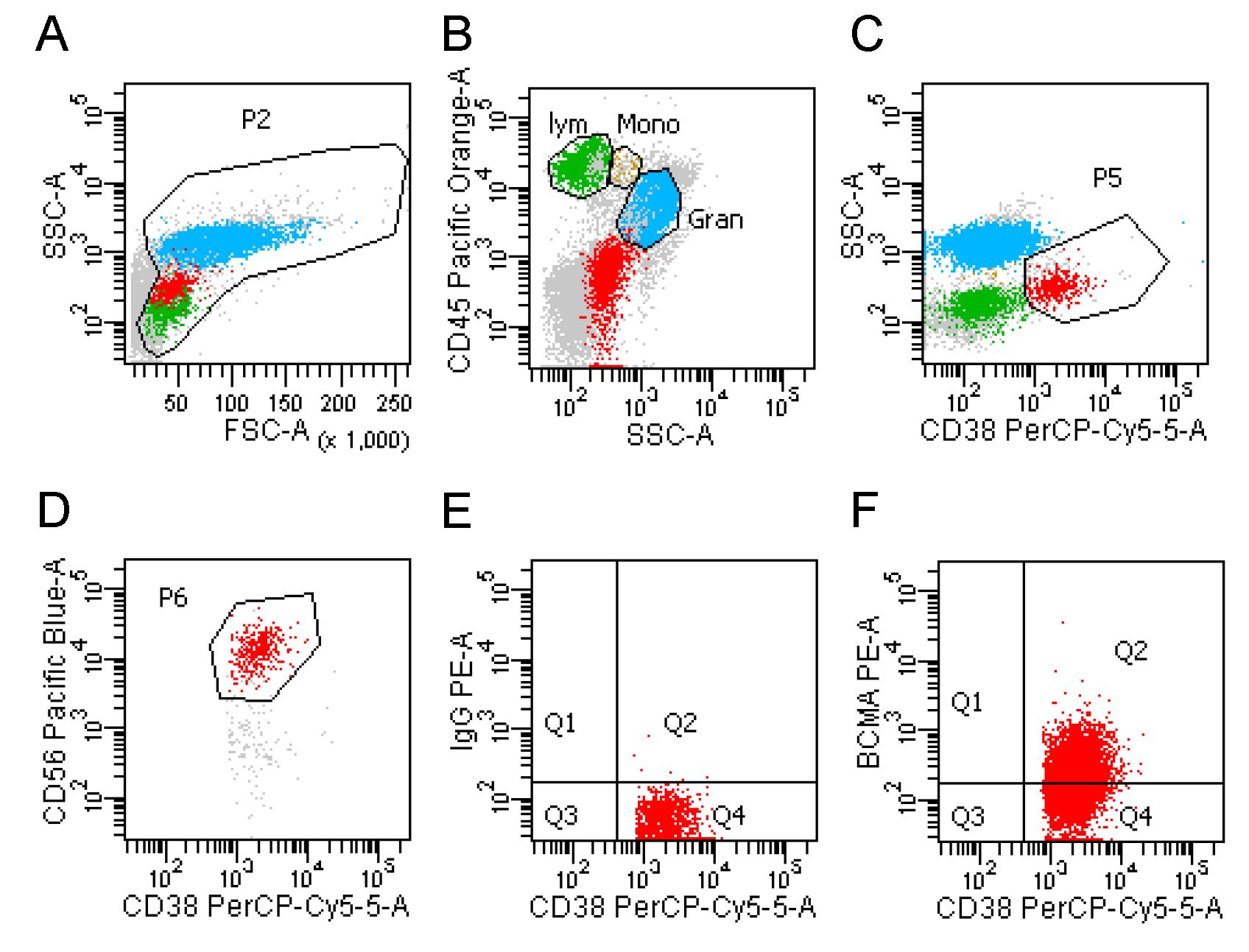


**Figure S1. The detailed gated method of flow cytometry.**

(A) The nucleated viable cells were gated by SSC and FSC. (B-D) CD45/CD38/CD56 were used to gate the abnormal plasma cells (P6). (E) Delineation of the crossover gate on the isotype control. (F) The BCMA positivity rate was calculated based crossover gate from isotype control. When the BCMA positivity rate was lower than 80-90%, the overall fluorescence intensity of abnormal plasma cells was too weak to distinguish the negative and positive cell clusters. Therefore, the result of MFI was calculated from the whole cluster of abnormal plasma cell entity, rather than the CD38+BCMA+ population.

**Figure S2.**

**
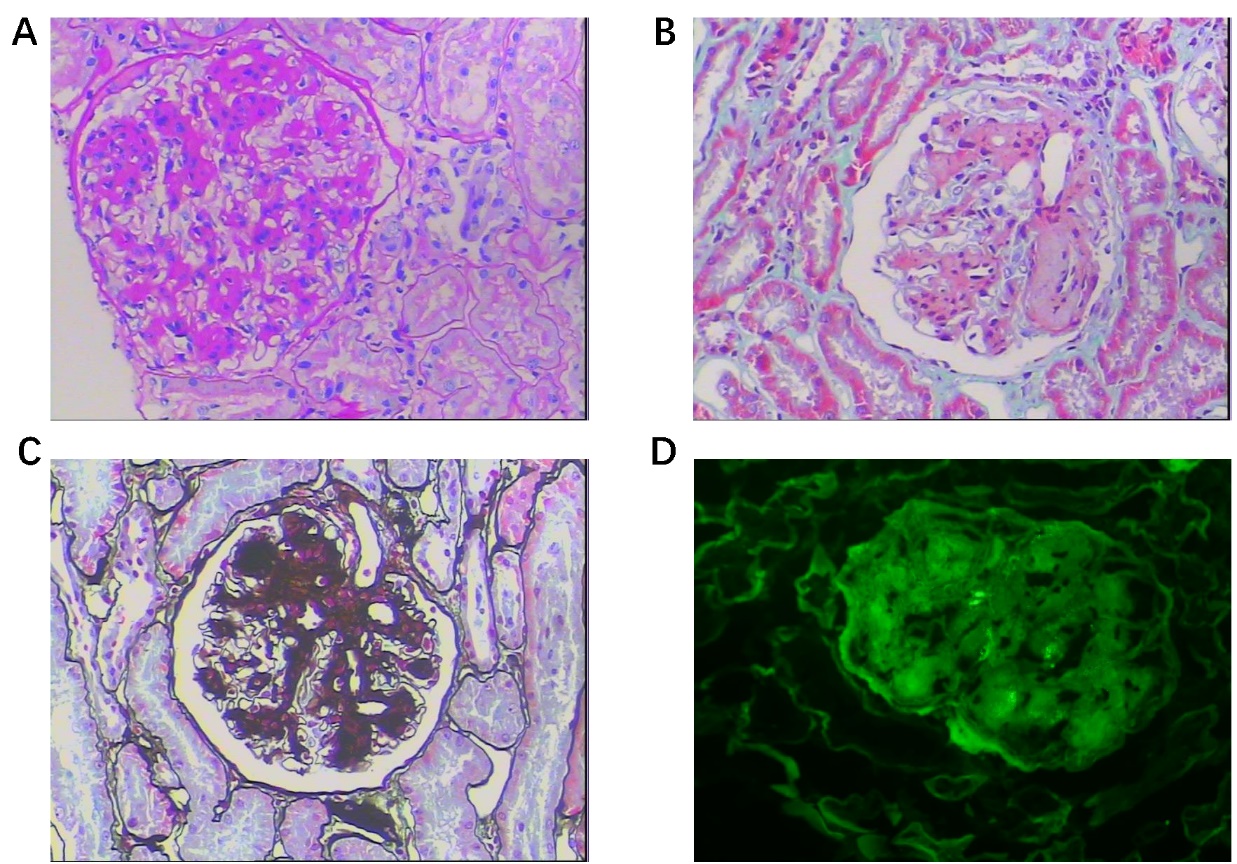
**

**Figure S2. The pathology of MGRS patient.**

(A-C) On light microscopy, the glomerulus shows marked global mesangial and segmental nodular mesangial sclerosis. The result of Congo red stain was negative. (B) On immunofluorescence, there is global granular mesangial staining for κ light chain. Glomeruli were negative for IgG, IgA, IgM, C3, C1q, FRA, and λ light chain.

Ultra-structurally, there are no obvious electron-dense deposits, and segmental subendothelial homogeneous mesangial deposits can be seen by electronic microscope (text-only result).

**Table S1. Spearman correlation analysis of clinical parameters with BCMA expression in** **newly diagnosed patients.**

|  | **BCMA positive rate** | | **BCMA MFI** | |
| --- | --- | --- | --- | --- |
|  | **Coefficient** | **P** | **Coefficient** | **P** |
| Gender | 0.048 | 0.428 | 0.028 | 0.642 |
| Age | 0.044 | 0.464 | -0.011 | 0.858 |
| Hemoglobin | -0.258 | < 0.001* | -0.088 | 0.141 |
| Serum albumin | 0.011 | 0.858 | 0.009 | 0.887 |
| Serum calcium | 0.107 | 0.076 | -0.037 | 0.540 |
| Serum creatinine | 0.053 | 0.378 | 0.021 | 0.728 |
| Serum β2-M | 0.141 | 0.042* | 0.058 | 0.410 |
| Serum LDH | 0.026 | 0.659 | 0.095 | 0.112 |

Abbreviations: β2-M, β2-microglobulin; LDH, lactic dehydrogenase; BCMA, B cell maturation antigen; MFI, mean fluorescence intensity; Auto-HSCT, autologous hematopoietic stem cell transplantation.

*: *p* values less than 0.05 (two-tailed) were considered statistically significant

**Table S2. Multivariate linear regression analysis of clinical parameters with BCMA expression in newly diagnosed patients.**

|  | **BCMA positive rate** | | | **Log BCMA MFI** | | |
| --- | --- | --- | --- | --- | --- | --- |
|  | **Coefficient** | **95% CI** | **P** | **Coefficient** | **95% CI** | **P** |
| Gender | 0.956 | -5.853 - 7.766 | 0.782 | 0.052 | -0.051 - 0.155 | 0.321 |
| Age | -0.186 | -0.551 - 0.179 | 0.315 | -0.006 | -0.011 - 0 | 0.035* |
| Hemoglobin | -0.334 | -0.476 - -0.192 | < 0.001* | -0.003 | -0.005 - -0.001 | 0.003* |
| Serum albumin | 0.578 | 0.095 - 1.06 | 0.019* | 0.005 | -0.002 - 0.013 | 0.157 |
| Serum calcium | 7.704 | -0.121 - 15.53 | 0.054 | 0.051 | -0.068 - 0.169 | 0.398 |
| Serum creatinine | 0.006 | -0.028 - 0.039 | 0.743 | < 0.001 | 0 - 0.001 | 0.145 |
| Serum β2-M | -0.096 | -0.646 - 0.453 | 0.73 | -0.004 | -0.012 - 0.005 | 0.369 |
| Serum LDH | 0.031 | -0.004 - 0.065 | 0.079 | 0.001 | 0 - 0.001 | 0.011* |

Abbreviations: β2-M, β2-microglobulin; LDH, lactic dehydrogenase; BCMA, B cell maturation antigen; MFI, mean fluorescence intensity; Auto-HSCT, autologous hematopoietic stem cell transplantation.

*: *p* values less than 0.05 (two-tailed) were considered statistically significant
